# Death of Dr. Bright

**Published:** 1859-02

**Authors:** 


*Death of Dr. Bright.*--London papers announce the death of Dr. Richard Bright, the eminent Physician, by which the medical profession has lost one of its most illustrious members. Dr. Bright died on December 16th, after a short illness. The lamented gentleman received patients and was out in his carriage on the previous Saturday, after which he complained of indisposition, and retired to his chamber, which he was destined never to leave again alive. Dr. Bright was the third son of Mr. Richard Bright, of Ham Green, Somerset, and was born in Bristol, in September, 1789, so that he was in his 70th year. The late Dr. Bright had contributed largely to the advancement of medical science by his numerous works was physician extraordinary to the Queen, was a fellow of the Royal Society and several other scientific institutions.

